# Supplementary material for: A plasmid system with tunable copy number
Source: Nat Commun. 2022 Jul 7;13:3908. doi: 10.1038/s41467-022-31422-0 (PMC9263177; doi:10.1038/s41467-022-31422-0)
Supplement: Supplementary file 1 — Supplementary Information [file 41467_2022_31422_MOESM1_ESM.pdf]

Supplementary Materials

MOTHER MACHINE

Cell morphology

In Supplementary Fig. S1, we compare histograms of cell morphologies for all aTc conditions. We observe that the cells are longer at $10\,\mathrm{ng\,mL^{-1}}$ aTc than $0.01\,\mathrm{ng\,mL^{-1}}$. However, the volume - surface area ratio is conserved.

Data selection

In order to discard pathological cells as well as detection errors the following threshold are used to select data.

1. $0.7\,\mu\mathrm{m} < D < 4.3\,\mu\mathrm{m}$
2. $0.005\,\mathrm{min^{-1}} <$ growth rate $< 0.04\,\mathrm{min^{-1}}$
3. $1.7\,\mu\mathrm{m} < L < 17\,\mu\mathrm{m}$
4. intensity$>$ 122 A.U.

The intensity threshold is set just a few units above the average background (110-120). The most restrictive condition is the one on growth rate. It is used to set apart non growing cells as well as cells that can not be tracked overtime due to detection errors.

Supplementary Fig. S8 shows histogram of cell properties before and after selection for one of the worst cases at $0.01\,\mathrm{ng\,mL^{-1}}$. The peak at 0 corresponds to cells without measured growth rate. Detection and tracking error may lead to cell tracks whose length decreases overtime leading to negative growth rate.

Stabilization of PCN

In Fig. S7, we plot cell properties as a function of time for each aTc concentration.

The number of detected cells quickly increases when cells populate the chambers. Then, the total number of detected cells decreases for all aTc conditions. This is largely due to chambers being filled by pathological cells that upon death block their chamber. Pathological cells mostly suffer from plasmid loss at low aTc while plasmid overload dominates at high aTc. We observe that plasmid loss is more prone to block chambers. Indeed, the more aTc –i.e. the higher the PCN– the longer cells survive in the chamber.

For aTc concentration of $1\,\mathrm{ng\,mL^{-1}}$ and above, we observe a stabilization of the average cell intensity after about 600 minutes (about 12 generations). We also observe a stabilization in growth rate around this time. Only the data points past this 600 min threshold are used in Fig. 3B and 3C of the main text, as well as Supplementary Fig. 4, for aTc conditions of $1\,\mathrm{ng\,mL^{-1}}$ and above.

For aTc concentration of $0.1\,\mathrm{ng\,mL^{-1}}$ and below, plasmid loss is too frequent, we are not able to measure a stabilized average cell intensity. We use all time points available for those concentrations in other plots across the manuscript.

Growth rate

Growth rate measurement particularly lacks precision for the low aTc conditions. Considering aTc concentration of $1\,\mathrm{ng\,mL^{-1}}$ and above we observe a constant growth rate with a 10% decrease for the highest aTc condition of $100\,\mathrm{ng\,mL^{-1}}$.

PCN distribution

In Supplementary Fig. S9, we plot fold change histograms and corresponding gamma distributions:

$$p(x) = \frac{1}{b^a \Gamma(a)} x^{a-1} e^{-\frac{x}{b}} \;, \tag{1}$$

with Γ the gamma function, $a = \frac{k_1}{\gamma_2} = \frac{\langle x \rangle^2}{\sigma^2}$ the mean number of burst per cycle (Friedman PRL 2006) and $b = \frac{k_2}{\gamma_1} = \frac{\sigma^2}{\langle x \rangle}$ the mean burst size.

MASSIVELY PARALLELIZED ASSAY

No Relationship Between Priming Promoter Strength and Plasmid Copy Number

Given that we saw a clear relation between promoter de-repression and plasmid copy number (Fig. 1D) and that high-copy number plasmids had similar origins of replication, we were surprised that we could not find a clear relation between the predicted strength [15] of the promoter controlling RNA-p and the plasmid copy number (Supplementary Fig. S3).

TABLE I.

Supplementary Table 1: Promoter Sequences, Next-generation sequencing counts, Relative Growth Rates, Predicted Plasmid Copy Numbers, and Predicted Promoter Strength for priming RNA variants used in this work

TABLE II.

Supplementary Table 2: Promoter Sequences, Next-generation sequencing counts, Relative Growth Rates, Predicted Plasmid Copy Numbers, and Predicted Promoter Strength for inhibitory RNA variants used in this work

TABLE III.

Supplementary Table 3: Sequencing Counts at each time point for priming RNA variants

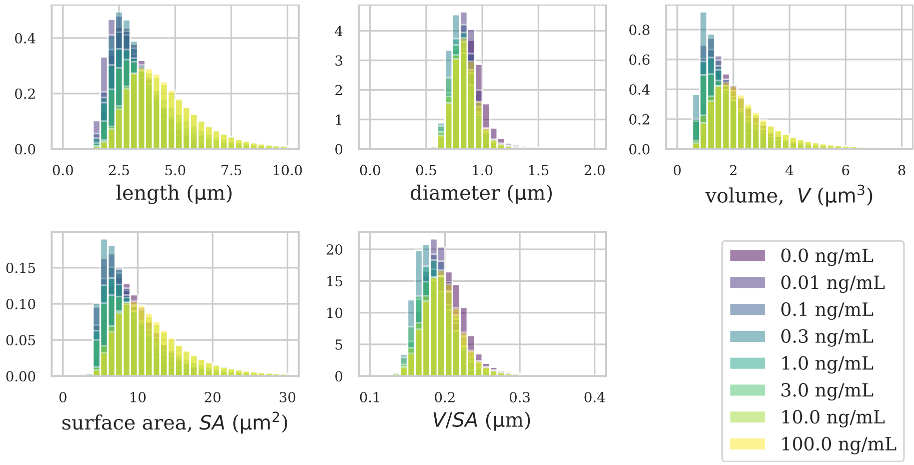

Fig. S1. Increase of cell length with aTc concentration, i.e. plasmid copy number, but conserved volume/surface area ratio.

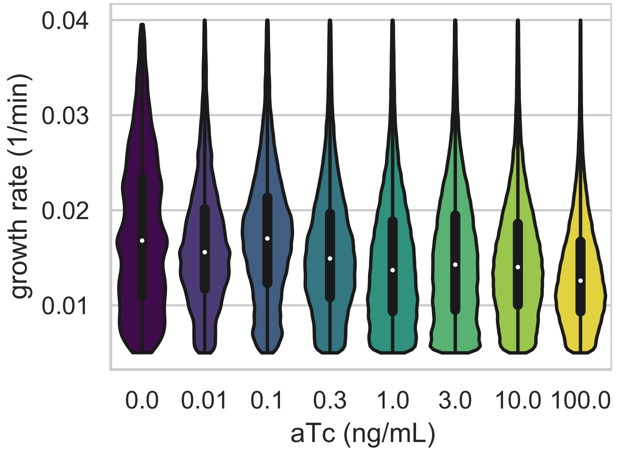

Fig. S2. Violin plots illustrating the distribution of growth rates for all aTc concentrations after selection. Upper and lower edges of center boxes represent first and third quartiles, center represents median. Maxima and minima are represented by ends of the whiskers. (c.f. Supplementary Fig. 1).

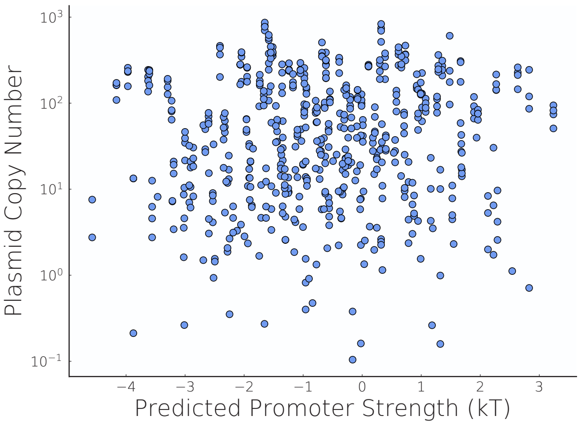

Fig. S3. Priming Promoter Strength vs Plasmid Copy Number

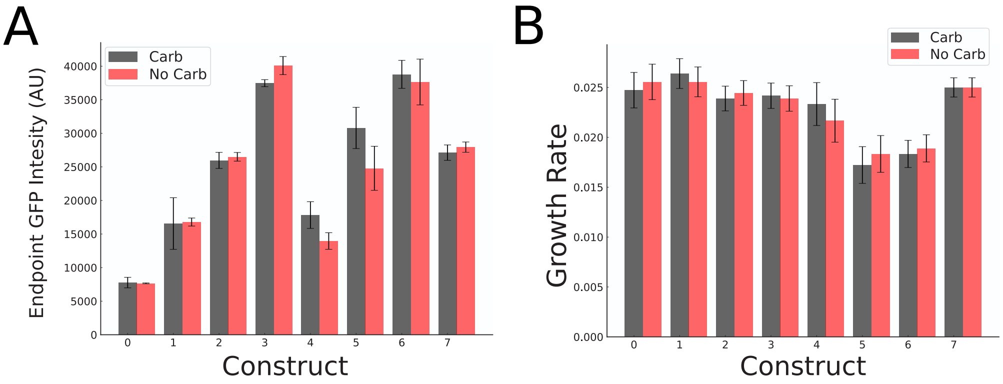

Fig. S4. Impact of antibiotic exposure on tunable plasmid copy number system. A) Endpoint sfGFP Fluorescence for constructs with and without antibiotic treatment. B) Growth Rates of constructs with and without antibiotic treatment

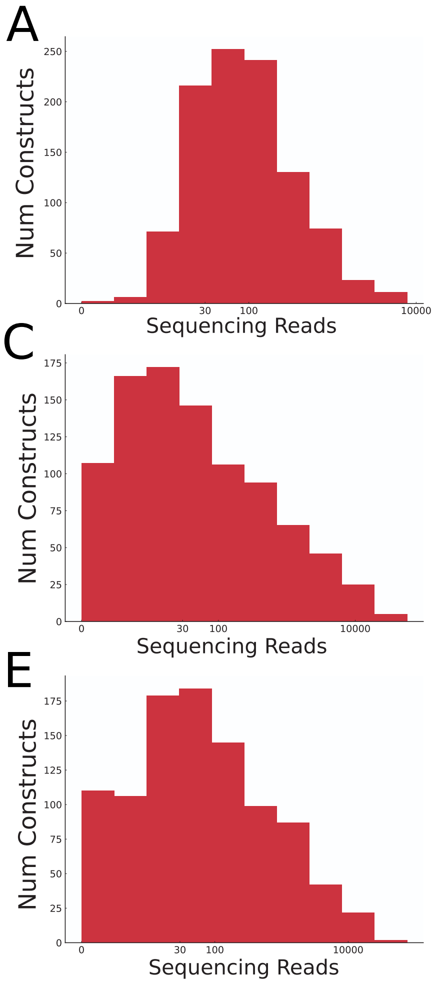

Fig. S5. Distribution of sequencing Reads at for A) Ligation Product, B) Time point 1, C) Time Point 2, D) Time Point 3, and E) Time Point 4

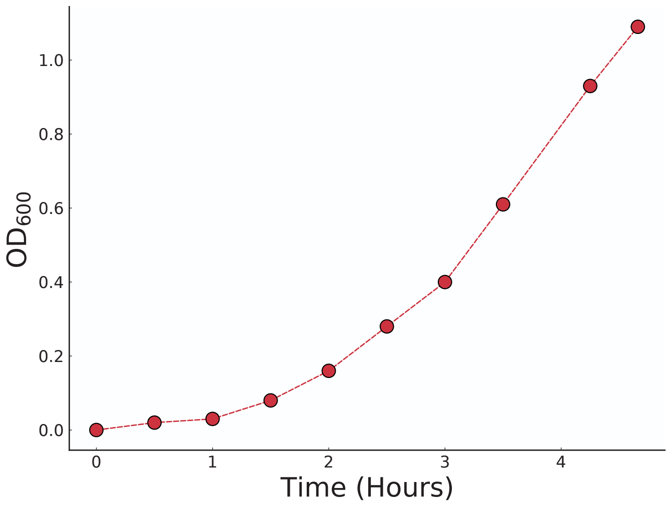

Fig. S6. Growth curve of liquid cultures hosting the priming promoter librabry in TB. Cells remain in the exponential phase through an $\mathrm{OD}_{600}of1.0$

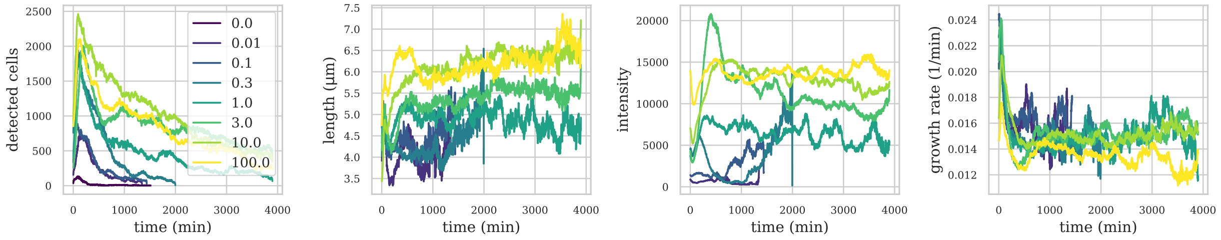

Fig. S7. Time dependent measurement of the number of detected cells, their averaged length, fluorescence and growth rate.

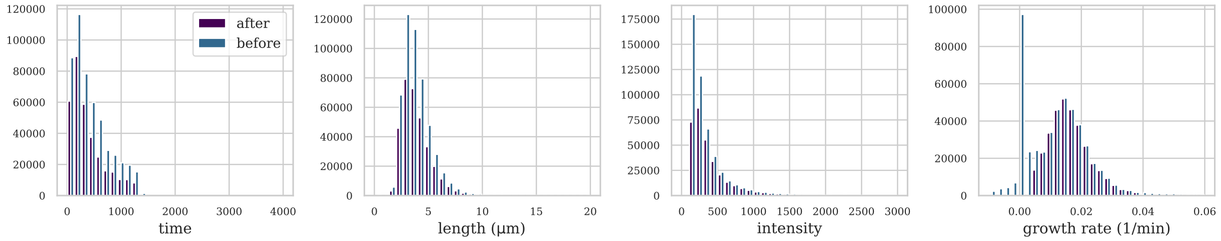

Fig. S8. Count of cells as a function of time, length, fluorescence intensity, and growth rate before and after selection for one of the most difficult condition at $0.01\,\mathrm{ng\,mL^{-1}}$ aTc.

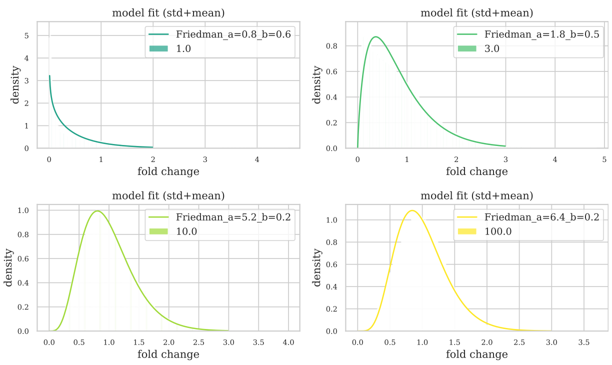

Fig. S9. Fit of fold change histograms for stabilized movies at 1, 3, 10 and $100\,\mathrm{ng\,mL^{-1}}$ aTc.
