# Supplementary material for: A plasmid system with tunable copy number
Source: Nat Commun. 2022 Jul 7;13:3908. doi: 10.1038/s41467-022-31422-0 (PMC9263177; doi:10.1038/s41467-022-31422-0)
Supplement: Supplementary file 2 — Description of Additional Supplementary Files [file 41467_2022_31422_MOESM2_ESM.pdf]

Title: Supplementary Data 1:
Description: Promoter Sequences, Next-generation sequencing counts, Relative Growth Rates, Predicted Plasmid CopyNumbers, and Predicted Promoter Strength for priming RNA variants used in this work

Title: Supplementary Data 2:
Description: Promoter Sequences, Next-generation sequencing counts, Relative Growth Rates, Predicted Plasmid Copy Numbers, and Predicted Promoter Strength for inhibitory RNA variants used in this work

Title: Supplementary Data 3:
Description: Sequencing Counts at each time point for priming RNA variants

Title: Supplementary Movie 1:
Description: Timelapse movie showing an increase in the fraction of elongated cells at high aTc levels.
